# Enhanced dynamics in the anomalous melting regime of DMPG lipid membranes

**DOI:** 10.1063/4.0000031

**Published:** 2020-10-16

**Authors:** Elizabeth G. Kelley, Michihiro Nagao, Paul D. Butler, Lionel Porcar, Bela Farago

**Affiliations:** 1 NIST Center for Neutron Research, National Institute of Standards and Technology, Gaithersburg, Maryland 20889, USA; 2 Center for Exploration of Energy and Matter, Indiana University, Bloomington, Indiana 47408, USA; 3 Department of Physics and Astronomy, University of Delaware, Newark, Delaware 19716, USA; 4 Department of Chemical and Biomolecular Engineering, University of Delaware, Newark, Delaware 19716, USA; 5 Department of Chemistry, The University of Tennessee, Knoxville, Tennessee 37996, USA; 6 Institut Laue-Langevin (ILL), Grenoble F-38042, France

## Abstract

Like many soft materials, lipids undergo a melting transition associated with a significant increase in their dynamics. At temperatures below the main melting transition (*T_m_*), all molecular and collective dynamics are suppressed, while above *T_m_* the alkyl tail motions, lipid diffusivity, and collective membrane undulations are at least an order of magnitude faster. Here we study the collective dynamics of dimyristoylphosphatidylglycerol (DMPG, di 14:0 PG) using neutron spin echo spectroscopy throughout its anomalous phase transition that occurs over a 12 °C–20° C wide temperature window. Our results reveal that the membranes are softer and more dynamic during the phase transition than at higher temperatures corresponding to the fluid phase and provide direct experimental evidence for the predicted increase in membrane fluctuations during lipid melting. These results provide new insights into the nanoscale lipid membrane dynamics during the melting transition and demonstrate how these dynamics are coupled to changes in the membrane structure.

## I. INTRODUCTION

Phospholipids undergo a main melting transition (*T_m_*) associated with a change in the lipid physical state from an ordered phase at low temperatures to a disordered and fluid phase at high temperatures. Usually, this transition is highly cooperative and occurs at a well-defined temperature that depends on the hydrophobic fatty acyl tail length and unsaturation as well as the hydrophilic headgroup structure.^1^ However, the phase behavior of lipids with charged phosphatidylserine (PS) and phosphatidylglycerol (PG) headgroups is more complex and can depend on the pH and ionic strength of the surrounding solution.^2^ The phase behavior of PG lipids is particularly unusual in buffers with low ionic strengths. The melting transition of phosphatidylglycerol lipids with saturated hydrocarbon chains ≤15 carbons broadens with decreasing tail length but is highly cooperative for tail lengths ≥16 carbons (dipalmitoylphosphatidylglycerol, DPPG) at low ionic strengths of the surrounding medium.^3^ Dimyristoylphosphatidylglycerol (DMPG, diC 14:0 PG) is especially well-studied because the melting transition spans over a 20 °C wide temperature window at low buffer ionic strengths and lipid concentrations and sharpens with increasing lipid and/or salt concentration in the buffer. What makes this transition even more unusual is that it is also associated with macroscopic changes in the lipid solution properties including the solution turbidity, viscosity, and conductivity.^3–5^

In general, the lipid melting transition is associated with a peak in membrane properties such as dye binding,^6^ ion permeability,^7,8^ and the swelling of multilamellar bilayers.^9–11^ It is thought that this peak arises from increased area and volume fluctuations in the membrane that are coupled with enthalpic fluctuations at the phase transition.^12–14^ However, the transition is usually only a few degrees wide (≲2 °C) in most phospholipids and is not associated with macroscopic changes in the solution properties as seen in DMPG solutions with a low ionic strength.

The peculiar behavior in DMPG solutions has inspired decades of work to understand the nanoscale changes in the membrane that lead to macroscopic solution property changes.^3–5,15–35^ Early works suggested that DMPG formed a sponge phase during the melting transition,^3,4^ while numerous follow-up studies have shown that the formation of an extended phase is unlikely. In particular, significant efforts by Riske, Lamy, and colleagues using a number of biophysical characterization techniques suggest that DMPG forms a highly perforated, porous membrane during the melting transition.^5,26–35^

While it is widely accepted that small (≈1 nm) transient pores form during the melting of zwitterionic phosholipids,^7,12^ several studies suggest that the pores formed in the DMPG melting regime may be larger and more stable. Spinozzi *et al.* reported the appearance of a correlation peak in small angle x-ray scattering (SAXS) data that shifts with temperature.^24^ The peak was attributed to in-plane correlations between the pores and their SAXS data model suggested that the pores were ≈15 nm in radius. These large pores are thought to be stabilized by changes in the spontaneous curvature in DMPG bilayers,^24,25,31,36^ similar to the perforated membranes formed by surfactant mixtures or mixtures of lipids and micelle-forming surfactants.^23,37^ Additional studies by Alakoskela *et al.* intentionally altered the spontaneous curvature of DMPG bilayers by incorporating small molecule additives and showed that the intermediate phase was stabilized by additives with a positive spontaneous curvatures, while additives with negative spontaneous curvature had the opposite effect.^23^

Forming the proposed perforated structures would be facilitated by a significant increase in the collective membrane fluctuations during the main melting transition. Riske *et al.* speculated that the enhanced area fluctuations associated with lipid melting could help stabilize and dramatically increase the fraction of pores in DMPG membranes.^31^ Yet the expected increase in dynamics has not been measured for this interesting lipid system because traditional tools used to characterize the membrane fluctuations and rigidity will not work. The loss of phase contrast during the DMPG phase transition prevents the use of methods that rely on optical microscopy of giant unilamellar vesicles (GUVs) such as micropipette aspiration, tether pulling, or fluctuation spectroscopy,^30^ while the high charge and associated strong electrostatic interactions make it difficult to form well-defined multilamellar stacks needed for diffuse x-ray scattering.^20,27^

Here we directly measure the collective membrane fluctuations on the nanometer length scales and nanosecond timescales throughout the phase transition in extruded DMPG vesicles using neutron spin echo spectroscopy (NSE). Our results show that the local membrane dynamics are enhanced, quantified as a decrease in relaxation time and associated softening of the membrane at temperatures corresponding to the anomalous phase transition. The behavior is seen in two lipid concentrations in the same low ionic strength buffer and suggests that softening is a characteristic of the lipid phase transition, as the membrane is even softer during the phase transition than at higher temperatures corresponding to the lipid fluid phase. We combine the NSE results with small angle neutron scattering (SANS) measurements of the bilayer structure to provide additional evidence of the enhanced area and thickness fluctuations that would be needed to form a highly perforated and/or porous membrane suggested by previous works. At the same time, the extreme degree of softening suggests that the dynamics are affected by the presence of the pores. Together, these results highlight that neutron scattering methods, and in particular, NSE, can be a useful complement to other biophysical characterization tools and provide important insights into the local membrane dynamics on the length scales of the membrane itself.

## II. METHODS

### A. Materials

Dimyristoylphosphatidylglycerol (DMPG) was purchased from Avanti Polar Lipids and used without purification. Deuterium oxide (D_2_O, 99.9%D) was purchased from Cambridge Isotopes, and all other chemicals were purchased from Sigma Aldrich and used as received. DMPG was dispersed in 10 mM 4-(2-hydroxyethyl)-1-piperazineethanesulfonic acid (HEPES) buffer containing 2 mM NaCl (pD 7.4) at a concentration of 1.8 percent by mass (wt. %) corresponding to 0.03 mol/L (M).

The lipid solutions were hydrated at 50 °C and then extruded through a 100 nm filter 31 times to produce a solution of relatively monodisperse lipid vesicles. All solutions were stored at temperatures well above the transition region until measurement and used within 1 day of preparation. Data were collected on cooling.

### B. Lipid phase behavior

The transition region of the DMPG vesicle solutions was determined using differential scanning calorimetry (DSC) and densitometry experiments. DSC experiments were performed using a Microcal VP-DSC calorimeter or Setaram MicroDSC III. The lipid solution was equilibrated at 60 °C, and data were measured on cooling using a scan rate of 0.5 °C/min. The buffer background and baseline corrections were performed in the software package provided with the instruments.

Densitometry measurements were performed on an Anton PaarDMA 5000 density meter. The lipid volume as a function of temperature was calculated following procedures in literature.^38^ The transition region was also determined from the derivative of the measured density vs temperature curve.^1^

### C. Membrane structure

The DMPG membrane structure was studied as a function of temperature using small angle neutron (SANS) scattering. SANS data were collected on the NGB30 SANS instrument at the National Institute of Standards and Technology (NIST) Center for Neutron Research (NCNR) and the D22 SANS instrument at the Institut Laue Langevin (ILL). Samples were run in 1 mm Quartz Cuvettes, and data were collected on cooling. The scattering data were reduced to absolute intensity using the IGOR Macros provided by NIST^39^ (NGB30 SANS) or GRASP^40^ (D22 SANS) and analyzed in SasView.^41^

### D. Membrane dynamics

DMPG membrane dynamics for the 30 mM solution were measured on the NGA NSE spectrometer at the National Institute of Standards and Technology (NIST) Center for Neutron Research (NCNR). Neutron wavelengths of 0.8 nm^–1^ and 1.1 nm^–1^ and a wavelength spread of Δλ/λ≈0.2 were used to access a *q*-range of 0.45 nm^–1^–1.2 nm^–1^ and Fourier times, *t*, range from 0.01 ns to 100 ns. The magnitude of the scattering vector, *q*, is defined as q=4π/λ sin θ/2 in which *θ* is the scattering angle. Data for the 3 mM solution were measured on the IN15 NSE spectrometer at the Institut Laue Langevin (ILL) using neutron wavelengths of 1 nm^–1^ and 1.35 nm^–1^ to access a *q*-range of 0.22 nm^–1^–1.1 nm^–1^ and to reach a maximum Fourier time of 400 ns.

The lipid solutions were equilibrated for at least 15 min before starting the measurement at the respective temperature. The temperature was controlled within 0.1 °C and data were collected on cooling. The data were corrected for the instrument resolution and buffer background using the DAVE software package for the NGA-NSE data^42^ and the procedures provided by ILL for the IN15-NSE data to give the intermediate scattering function (ISF).

The ISF data were fit as described in the main text. The diffusion coefficient, *D*, in Eq. (1) was determined from independent dynamic light scattering (DLS) measurements and fixed during the NSE data analysis. Temperature-dependent DLS data were either collected offline using a Wyatt NanoStar Pro or inline with a fiber optic DLS on the IN15-NSE. The sample path length was minimized for the offline DLS measurements to minimize the effects of multiple scattering on the estimates of *D* for the 30 mM DMPG sample. Measurements were performed with the small volume disposable cuvettes with a 3 mm sample path length in the Wyatt NanoStar Pro. However, it is important to note that the multiple scattering could result in an apparent increase in *D*, particularly in the gel and fluid phases where the samples are more turbid. As a result, the values for ⟨τ⟩ and $\tilde{\kappa }$ determined from fits to the NSE data may be too high. For example, a 10% increase in *D* due to multiple scattering would result in an ≈20% increase in ⟨τ⟩ and $\tilde{\kappa }$.

## III. RESULTS

### A. DMPG membranes have a wide transition region that depends on lipid concentration

**FIG. 1.**
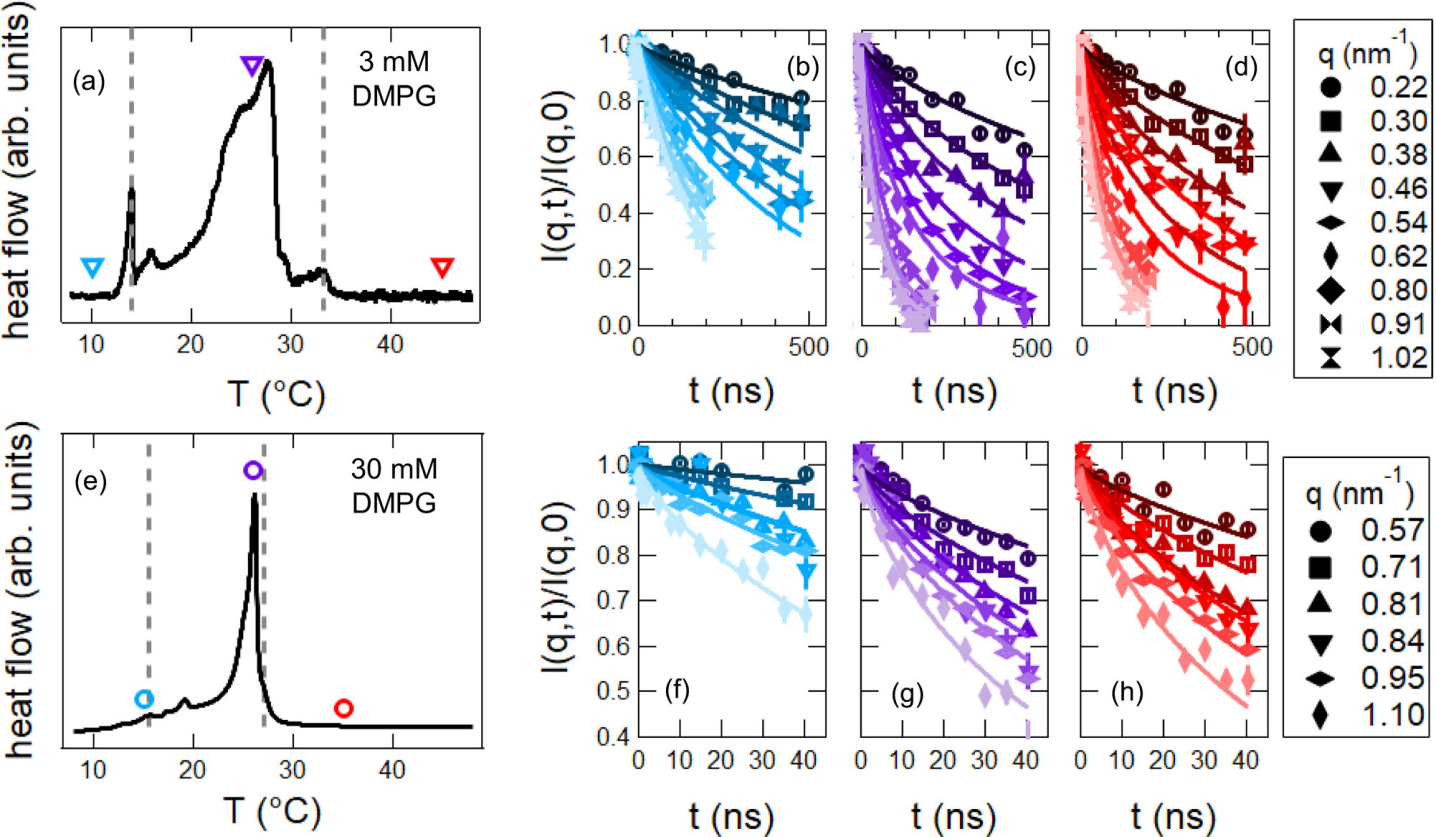
Phase behavior and representative intermediate scattering functions measured for the different lipid phases for (top) 3 mmol/L (mM) and (bottom) 30 mM DMPG vesicle solutions in a D_2_O buffer containing 10 mM HEPES and 2 mM NaCl, pD 7.4. The dashed lines in the differential scanning calorimetry (DSC) traces in (a) and (e) represent the onset ($T_{n}^{on}$) and end point ($T_{m}^{\mathrm{off}}$) of the phase transition. Temperatures between these limits correspond to the intermediate phase. The colored symbols in (a) and (e) correspond to the temperature points of the representative NSE data presented in (b)–(d) for the 3 mM and (f)–(g) for the 30 mM DMPG solutions. The blue, purple, and red colors correspond to the gel phase, transition region, and fluid phase, respectively. Error bars represent one standard deviation throughout the manuscript and are smaller than the data symbols in some cases.

Figures 1(a) and 1(e) show the differential scanning calorimetry (DSC) traces measured for 3 mmol/L (mM) and 30 mM DMPG unilamellar vesicle solutions prepared in a D_2_O buffer solution containing 10 mM HEPES, 2 mM NaCl with pD = 7.4. Qualitatively the DSC traces look similar for the two lipid concentrations and show several broad features beginning around T ≈15 °C and ending around T ≈35 °C that correspond to the anomalous phase transition. Rather than defining a single temperature for the phase transition as is usually done for phospholipids, we use the nomenclature found in the DMPG literature and refer to the onset of the phase transition as $T_{m}^{on}$ and the end point as $T_{m}^{\mathrm{off}}$. Temperatures below $T_{m}^{on}$ correspond to the lipid gel phase, and above $T_{m}^{\mathrm{off}}$, the lipid fluid phase. We refer to temperatures between $T_{m}^{on}$ and $T_{m}^{\mathrm{off}}$ as the transition region, which is also referred to as the intermediate phase in some literature.

Quantitatively comparing the DSC traces for the two lipid concentrations shows that the phase transition is broader for the 3 mM solution with $T_{m}^{on}=14.0\;{}^{\circ}$C and $T_{m}^{\mathrm{off}}=33.3\;{}^{\circ}$C compared to $T_{m}^{on}=15.5\;{}^{\circ}$C and $T_{m}^{\mathrm{off}}=27.0\;{}^{\circ}$C for the 30 mM solution. The approximately 7 °C decrease in the width of the phase transition with an order of magnitude increase in lipid concentration is in good agreement with previous studies of DMPG in literature.^4,28,32^ The concentration dependence of the phase transition is thought to be related to the degree of ionization of the DMPG bilayer and effective changes in the ionic strength of the surrounding solvent, where the effective increase in the medium ionic strength with increasing lipid concentration leads to a more cooperative phase transition.^32^

Quite interestingly, while the measured $T_{m}^{on}$ and $T_{m}^{\mathrm{off}}$ temperatures for the extruded vesicles studied here were consistent with previously reported values for unextruded DMPG dispersions, the shapes of the DSC curves are different.^28,32^ DSC curves reported in the literature for DMPG usually show a sharp peak at a few degrees above $T_{m}^{on}$, which are not seen in the data in Fig. 1. The data in Fig. 1 look more similar to DSC curves reported in literature after multiple heating and cooling cycles.^34^ These subtle differences in the shape of the DSC curves suggest that sample preparation, storage temperature, and storage time could potentially affect shape of the transition region. For example, Epand *et al.* reported the formation of a metastable phase in DMPG suspensions stored at low temperatures for extended periods of time.^20^ As such, we were careful to use the same sample preparation procedure for all experiments here, and the same sample for complementary techniques whenever possible. All DMPG vesicles were extruded and stored above $T_{m}^{\mathrm{off}}$ and were used within one day of preparation. All experimental data were collected on cooling the sample from above $T_{m}^{\mathrm{off}}$ through transition region and below $T_{m}^{on}$.

### B. DMPG membrane dynamics are faster during the phase transition

We studied the collective membrane dynamics as a function of temperature in each region of the phase transition. Figures 1(b)–1(d) and 1(f)–1(h) show representative data for the intermediate scattering function (I(q,t)/I(q,0)) vs Fourier time (*t*) measured with NSE in each phase. The NSE data for the gel phase decay much slower than at higher temperatures, consistent with the expected slow dynamics. The curves decay faster for the transition region and fluid phases, indicative of the faster membrane undulations as the lipid melt.

The temperature-dependent changes in the membrane dynamics were quantified by fitting the intermediate scattering functions. The solid lines in Figs. 1(b)–1(d) and 1(f)–1(g) are fits to

$$I(q,t)/I(q,0)=\text{exp}\;[-Dq^{2}t]\;\text{exp}\;[-{\left(\Gamma_{b}(q)t\right)}^{2/3}],$$ (1)

in which the first term accounts for the diffusion of the vesicles with diffusion constant, *D*, that were measured with dynamic light scattering (DLS, see the Methods Section and supplementary material for more details).^43–45^ The second term is the stretched exponential function predicted by Zilman and Granek for membrane bending fluctuations with a relaxation rate Γ_*b*_.^46,47^ Γ_*b*_ was the only fit parameter. Equation (1) fits the data well, suggesting that the membrane dynamics in all three phases follow the same scaling seen in other phospholipid membranes in the literature.

**FIG. 2.**
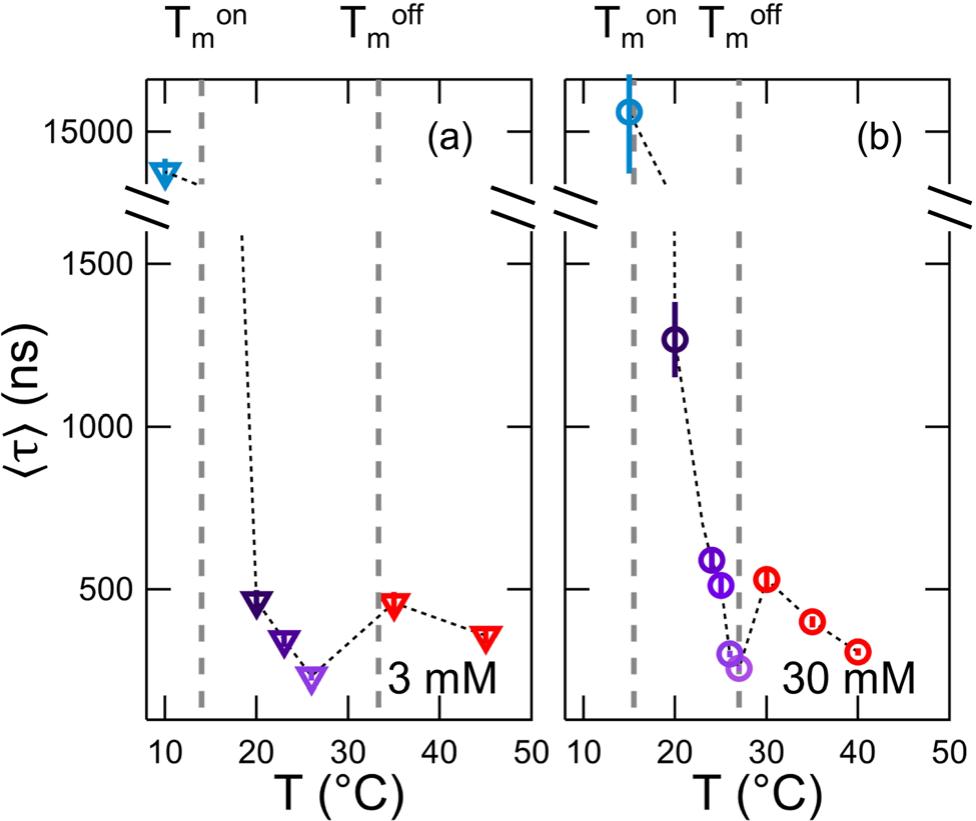
Average relaxation time, $\left\langle \tau \rangle =\frac{\tau }{\alpha }\Gamma (\frac{1}{\alpha }\right)$, determined from stretched exponential fits to the intermediate scattering functions for (a) 3 mM DMPG and (b) 30 mM DMPG at *q* = 0.8 nm^–1^ as a function of temperature. The y-axis has been split to show the increase in dynamics in the transition region (purple points) compared to the gel (blue points) and fluid phases (red points). The dotted line connecting the points is to guide the eye. Vertical dashed lines show the temperature range corresponding to the transition region for the different lipid concentrations.

The enhanced dynamics throughout the phase transition are clearly seen in a plot of average relaxation time (⟨τ⟩) vs temperature, where $\langle \tau \rangle =\frac{\tau }{\alpha }\Gamma (\frac{1}{\alpha }),\;\tau =1/\Gamma_{b}$, and $\Gamma (\frac{1}{\alpha })$ is a gamma function of the inverse of the stretching exponent, *α*. Here we calculated ⟨τ⟩ using *α* = 2/3 from Eq. (1). The fit results are shown in Fig. 2 for *q* = 0.8 nm^–1^. This *q* corresponds to length scales of ≈8 nm that are greater than the bilayer thickness (≈4 nm) and less than the vesicle radius (≈40 nm–50 nm) where the Zilman–Granek framework is applicable.^46,47^ The average relaxation time decreased by an order of magnitude above $T_{m}^{on}$ to <300 ns and then increased by more than 50% above $T_{m}^{\mathrm{off}}$ at both lipid concentrations. The same trend in ⟨τ⟩ was seen at all measured *q*-values (see supplementary material), with the average relaxation time decreasing with increasing *q*.

The fluctuation relaxation rate at these length- and timescales is dictated by a balance between the membrane stiffness and the bulk friction with the solvent. The model by Zilman and Granek predicts that Γ_*b*_ will follow a *q*^3^ dependence that is inversely related to the effective membrane stiffness, $\tilde{\kappa }$,

$$\Gamma_{b}=0.025\frac{k_{b}T}{\eta }\sqrt{\frac{k_{b}T}{\tilde{\kappa }}}q^{3},$$ (2)

where *η* is the solvent viscosity, T is the temperature, and *k_b_* is the Boltzmann's constant.^46,47^ Accordingly, the reduction in $\Gamma_{b}^{-1}$ seen in transition region suggests that the membranes are significantly softer and $\tilde{\kappa }$ was reduced.

**FIG. 3.**
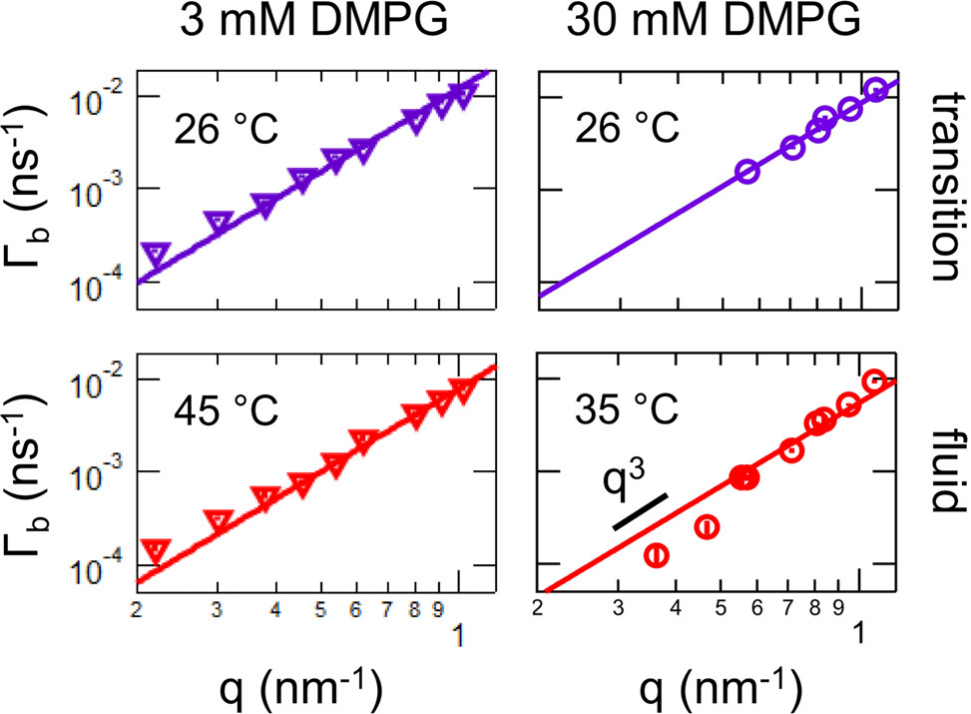
Log–log plot of the measured decay constants, Γ_*b*_, vs *q* for the transition region and fluid phase for (left) 3 mM and (right) 30 mM DMPG in D_2_O buffer containing 10 mM HEPES and 2 mM NaCl, pD 7.4. The temperatures of the measurements are indicated on the plots. The solid lines are the best fit to *q*^3^-scaling at high *q*.

We extracted values of $\tilde{\kappa }$ from plots of Γ_*b*_ vs *q*, as shown in Fig. 3. We focused on the temperatures corresponding to the fluid phase and transition region where the measured relaxation times fall within the instrument resolution, and the solid lines are the best fit to the predicted *q*^3^ scaling at high *q*. The data for both the transition region and fluid phase were well described by this scaling up to *q* ≈1.2 nm^–1^, suggesting that the soft membranes are undergoing the same thermal bending fluctuations seen in other fluid membranes in literature. We note that we did not measure the dynamics to higher *q*-values because these small length scales are similar to the bilayer thickness and the dynamics may be affected by the local molecular motions of the lipid molecules. Similarly, the dynamics at lower *q*-values may also be affected by other dynamic modes as Zilman and Granek's description of single membrane fluctuations is no longer valid at the length scale of the vesicle radius.^46,47^ The data measured at q ≲ 0.5 nm^–1^ begin to deviate from the *q*^3^ scaling predicted by Zilman and Granek possibly due to contributions from these other modes.

Several works in the literature have shown that the membrane stiffness measured at the short length scales and timescales of NSE is significantly higher than the values measured with techniques such as flicker spectroscopy and x-ray scattering methods due to the dissipation within the bilayer itself.^48–50^ In other words, the lateral lipid flow that allows the membrane height (bending) fluctuations to relax is suppressed by the viscous drag within a lipid leaflet as well as the friction between the two leaflets at the timescales measured with NSE.^45,50–52^ As such, $\tilde{\kappa }$ contains contributions from both the elastic bending modulus (*κ*) as well as dissipation within the bilayer. Watson and Brown accounted for this additional source of dissipation within lipid bilayers by incorporating Seifert and Langer model^53^ for membrane dynamics that includes monolayer density fluctuations into the Zilman–Granek framework for NSE data discussed above.^50^ Watson and Brown suggested that

$$\tilde{\kappa }=\kappa +2d^{2}k_{m},$$ (3)

where *κ* is the elastic bending modulus of a membrane, *d* is the height of the neutral surface, and *k_m_* is the monolayer compressibility modulus.^50^

**FIG. 4.**
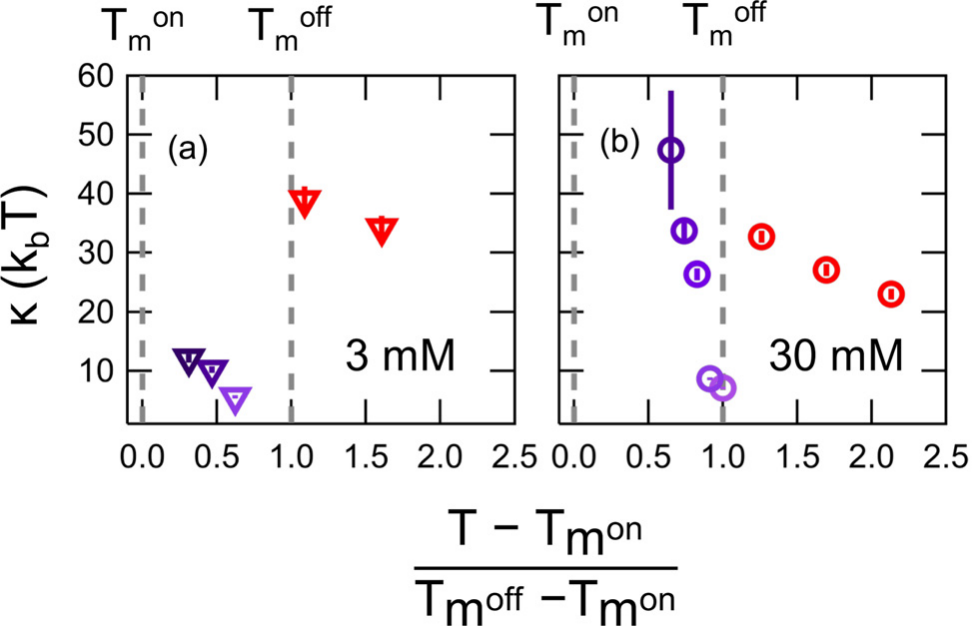
Bending modulus, *κ* determined from the NSE data for the transient region and fluid phase vs normalized temperature for (a) 3 mM and (b) 30 mM DMPG. The temperatures is normalized as $\left(T-T_{m}^{on})/(T_{m}^{\mathrm{off}}-T_{m}^{on}\right)$ such that 0 corresponds to the beginning of the transition region and 1 is the end point of the transition. Normalized temperature values >1 correspond to the fluid phase.

Using relationships for *d* and *k_m_* that assume the polymer brush model of lipid membranes is applicable^54^ as has worked well for other phospholipid membranes in the literature gives $\tilde{\kappa }\approx 13\kappa$,^45,50,51^ which means that the fluctuations relax at the nanoscale as though the membrane is effectively an order of magnitude stiffer and that the contributions from the internal dissipation are significant. From these relationships, we then calculated the temperature dependence of *κ* in the intermediate and fluid phases.^45,51,52,55,56^ These values are shown in Fig. 4 as a function of a normalized temperature for both the (a) 3 mM and (b) 30 mM DMPG solutions. The temperatures were normalized to account for the differences in width of the phase transitions between the two lipid concentrations, as $\left(T-T_{m}^{on})/(T_{m}^{\mathrm{off}}-T_{m}^{on}\right)$, such that 0 is the onset and 1 is the end point of the phase transitions. Near $T_{m}^{\mathrm{off}}$ *κ* was on the order of 10 k_*b*_T a value more typical of a single monolayer rather than a lipid bilayer. *κ* did not increase to values similar to those reported for zwitterionic phospholipid bilayers (30 k_*b*_T–40 k_*b*_T)^57,58^ until temperatures above $T_{m}^{\mathrm{off}}$.

### C. The bilayer thickness changes continuously through the phase transition

**FIG. 5.**
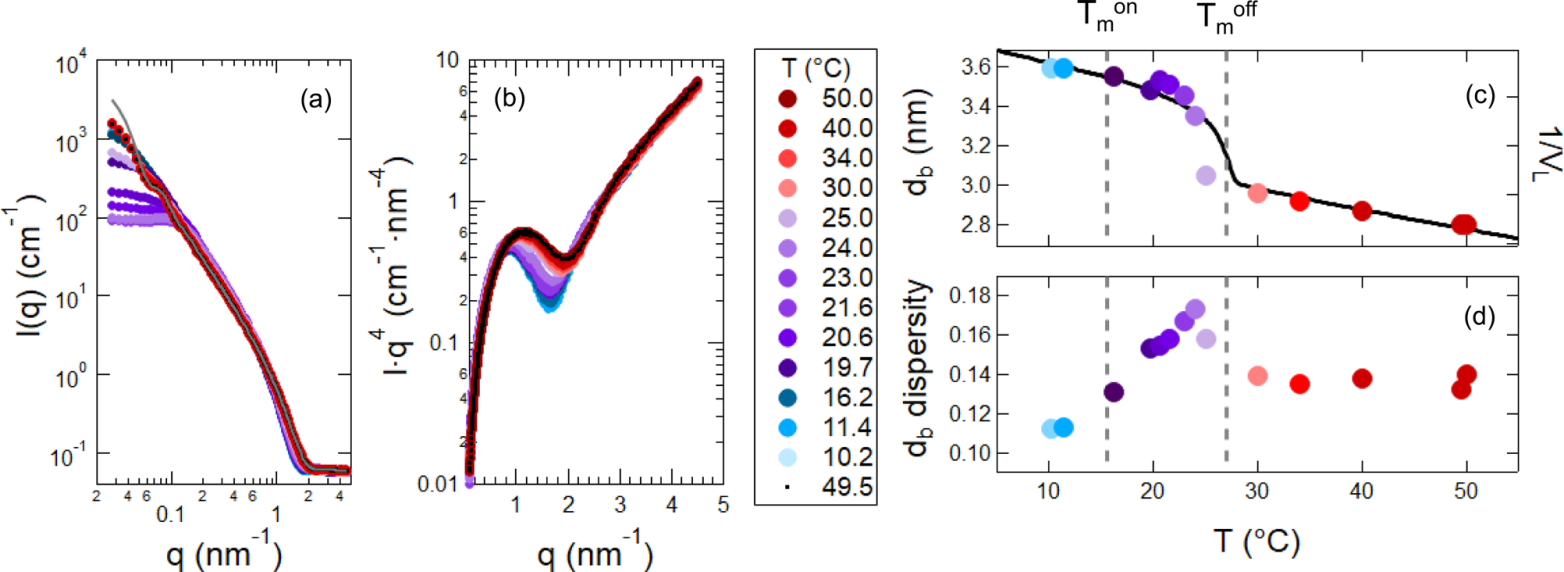
SANS data and fitting results for 30 mmol/l (mM) DMPG. Temperature series of SANS data collected plotted as (a) intensity *I*(*q*) vs *q* and (b) $I\cdot q^{4}$ vs *q*. SANS data were collected on cooling and then the samples was reheated to 49.5 °C as listed in the legend from top to bottom. The solid gray line in (a) is the vesicle form factor fit to the 50 °C data extrapolated to low *q*. All data were fit for q≥ 0.5 nm^–1^. Fit results for the (c) bilayer thickness (*d_b_*) and (d) dispersity. The solid line in (c) is 1/*V_lipid_* measured with densitometry. The dashed lines indicate $T_{m}^{on}$ and $T_{m}^{\mathrm{off}}$ and the blue, purple and red colored points correspond to the gel phase, transition region, and fluid phase, respectively.

While the membrane dynamics showed a non-monotonic trend and enhancement in the transition region, the bilayer structure appeared to follow a continuous transition. Shown in Figs. 5(a) and 5(b) are the small angle neutron scattering (SANS) data collected for 30 mM DMPG across the phase transition. The SANS data at high *q* were consistent with a bilayer structure throughout the temperature range, in good agreement with previous small angle x-ray scattering (SAXS)^24,27^ and nuclear magnetic resonance (NMR) results.^34^ The scattering curve was completely recovered after cooling the sample through the transition region into the gel phase and then reheating back to 50 °C.

The low *q* data showed the presence of a significant structure factor contribution due to the strong electrostatic interactions in the highly charged systems. Interestingly, the low *q* dropped significantly in the transition region, suggesting stronger repulsive interactions during lipid melting than in the gel or fluid phase. The more repulsive interactions are consistent with reports that showed the sodium ions dissociate from the PG headgroups during lipid melting.^32,34^ Unfortunately, the structure factor made it impossible to extract any information about changes in the overall vesicle size and dispersity as a function of temperatures.

As such, we focused on fitting the SANS data at high *q* (q≥ 0.5 nm^–1^ as in the NSE analysis) that correspond to the bilayer structure with a vesicle model. Because the vesicle radius is an order of magnitude greater than the bilayer thickness, its contribution to the scattering is constant at high *q*.^59^ Therefore, we fixed the overall vesicle size and dispersity during the fitting without affecting the fit parameters related to the bilayer structure. We fit the SANS data with a vesicle form factor that treated the bilayer as a slab of constant scattering length density (*ρ*) that was convoluted with a Gaussian to account for polydispersity in thickness due to the thermal fluctuations in bilayer thickness.^59,60^

Plotted in Fig. 5(c) is the bilayer thickness and in Fig. 5(d) is the width of the Gaussian determined from fitting the SANS data. While the bilayer thickness continuously decreased with increasing *T* throughout the phase transition following the changes in lipid volume (*V_L_*), the polydispersity did not. The fit values for polydispersity qualitatively follow the trends measured in the membrane dynamics [Fig. 5(d)], with the largest values in the transition region.

### D. DMPG membranes are more compressible in the intermediate phase

In addition to bending fluctuations, lipid membranes also undergo collective thickness fluctuations out of the plane of the membrane on the nanoscale.^61–63^ The amplitude of these fluctuations is governed by another membrane elastic property: the area compressibility modulus (*K_A_*).^45,51^ The compressibility modulus is most often measured by micropipette aspiration of GUVs;^54^ however, these studies are not possible in the DMPG transition region as mentioned in Sec. I. We have shown that measuring the collective thickness fluctuations with NSE is another means of determining *K_A_*,^45,51^ but unfortunately these measurements also were unsuccessful for DMPG. Measuring the thickness fluctuations requires contrast matching the lipid tails to the surrounding solvent to emphasize the scattering from the headgroups. While this method has worked well for lipids with zwitterionic phosphocholine (PC) headgroups ($\Delta \rho^{2}$ = 0.21 fm/Å^3^), the exchangeable hydrogens and low contrast between the PG headgroups and surrounding solvent ($\Delta \rho^{2}$ = 0.12 fm/Å^3^) resulted in too low of a signal to be measured with NSE.

**FIG. 6.**
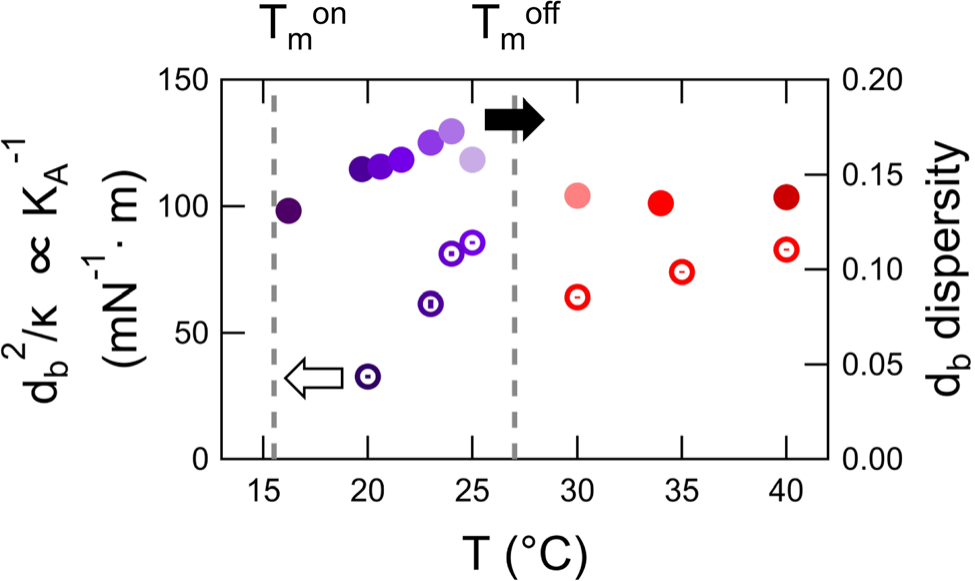
Estimated trends in the thickness fluctuation amplitude in the transition region and fluid phase. Bilayer thickness, *d_b_*, divided by *κ* determined by combining the NSE and SANS results for 30 mM DMPG (left axis, open symbols) where $d_{b}^{2}/\kappa$ is inversely proportional to the bilayer compressibility modulus, *K_A_*, and distribution of bilayer thickness determined from fitting the SANS data (right axis, closed symbols). Both $K_{A}^{-1}$ and *d_b_* dispersity are related to the amplitude of the collective thickness fluctuations. The dashed lines correspond to the onset and end point of the phase transition and the purple and red points correspond to values calculated for the intermediate and fluid phases, respectively.

While we were not able to directly measure the thickness fluctuations with NSE, we can get qualitative information about changes in *K_A_* upon DMPG melting from our NSE and SANS results discussed above. The bending modulus scales quadratically with bilayer thickness following the well-established relationship $\kappa /d_{c}^{2}=\beta K_{A}$ in which *d_c_* is the thickness of the hydrocarbon region of the bilayer and *β* is a constant. The ratio of *κ* measured with NSE and *d_b_* measured with SANS are presented for the 30 mM DMPG samples in Fig. 6. The results from these combined measurements suggested that *K_A_* drops by 30%–60% in the transition region compared to temperatures above $T_{m}^{\mathrm{off}}$, as also seen in Fig. 4 for *κ*.

Similarly, the increase in polydispersity in *d_b_* [Fig. 5(d)] determined from the SANS data modeling also pointed towards an increase in the amplitude of the membrane thickness fluctuations. The dispersity in bilayer thickness is attributed to the amplitude of thermal motions of the soft membranes,^64,65^ so the increase in *d_b_* dispersity in the transition region was also indicative of an ≈15%–30% increase in the collective fluctuation amplitude due to a decrease in *K_A_* during DMPG melting.

## IV. DISCUSSION

Lipid membranes undergo a wide array of dynamic fluctuations ranging for the rotation and diffusion of individual lipids to large scale membrane remodeling on the micrometer scale. At the mesoscale, the membranes undergo collective undulations on the local length scale of the membrane involving tens to hundred of lipids. The NSE results clearly show an increase in these collective dynamics at the mesoscale throughout the DMPG phase transition and provide direct experimental evidence for an increase in the local fluctuations. The scattering length density contrast between the protiated lipid membrane and deuterated buffer makes NSE sensitive to the local height fluctuations normal to the plane of the membrane over distances smaller than 10 nm. The amplitude of these fluctuations is inversely proportional to the membrane stiffness, $\kappa^{-1}$. Accordingly, the NSE data suggest that these fluctuations are both faster (decrease in ⟨τ⟩, Fig. 2) and larger in amplitude (decrease in *κ*, Fig. 4) during the phase transition.

The dramatic increase in dynamics in the transition region compared to the higher temperature fluid phase does not follow the traditionally expected trend with temperature. Because these motions are thermally excited, they should monotonically increase with thermal energy and increasing temperature above the phase transition. A continuous softening well-above *T_m_* has been measured for a wide-array of membranes composed of different lipids.^45,49,51,57^ Any anomalous changes in the membrane dynamics and associated elastic properties are only seen in the direct vicinity of the phase transition.^57,66^ The dynamics for DMPG membranes measured here directly mirror the trends seen in the excess heat capacity. The minimum in relaxation time (⟨τ⟩, Fig. 2) and bending modulus (*κ*, Fig. 4) occurs at ≈26 °C, the same temperature as the maximum in excess heat capacity measured with DSC in Figs. 1(a) and 1(e). This result is in excellent agreement with several previous works that show the increase in area fluctuations are related to the enthalpic fluctuations of the phase transition.^12–14^

While the enhanced membrane fluctuations are expected during the main melting transition, the extent of the softening is surprising for the highly charged DMPG membranes studied here. Recent work by Kuklin *et al.* showed a similar softening of dimyristoylphosphatidylcholine (DMPC, di 14:0 PC) lipid bilayers in the vicinity of the phase transition, a zwitterionic lipid with the same 14 carbon alkyl tails in DMPG studied here.^66^ They report a 33% decrease in *κ* over a <0.5 °C temperature range in DMPC as compared to ≈60%–70% decrease over a >10 °C window in DMPG in low ionic strength buffers seen here. The difference in temperature window is consistent with the well-documented melting behavior of the two different lipids; however, the ≈2× greater softening during the melting of DMPG compared to DMPC is unexpected, specifically with the significant increase in membrane charge in the DMPG intermediate phase.

The increased repulsion suggested by SANS data at low *q* [Fig. 5(a)], as well as seen in previous SAXS,^27^ solution conductivity,^32^ and NMR studies^34^ all indicate that DMPG membrane charge increases during the phase transition. A number of theoretical works suggest that increasing the charge density would make the membrane stiffer.^57,67–72^ The general rationale is that the fluctuations are suppressed to maximize the distance between the charged headgroups,^57,71^ which has been seen in experimental measurements of surfactant^73,74^ and lipid membranes^75^ with low surface charges. The DMPG membrane dynamics in the transition region show the exact opposite behavior. One possibility is that the presence of stable pores is significantly contributing to the apparent membrane softening. The presence of large pores throughout the membrane may cut off the long wavelength fluctuations that relax more slowly. Shifting the fluctuation distribution to shorter wavelengths would shorten the measured relaxation time which we would interpret as membrane softening in the current analysis framework. Alternatively, work by Prost *et al.* showed that membrane fluctuations can relax by permeation of water though the bilayer, and this effect dominates over the hydrodynamic process in porous membranes.^48,76^ While our NSE data seem to suggest that the collective membrane dynamics affect pore formation and vice versa, there could also be other dynamic contributions we have not even considered and additional studies are needed to better understand relaxation mechanisms in porous membranes.

The highly charged nature of the membranes in the transition region could also lead to correlated charge density fluctuations at the membrane surface.^77,78^ A few theoretical works suggest that such charge density fluctuations could lead to a softening of highly charged membranes in low salt buffers.^77,78^ While the present work focused on measuring the predicted increase in local fluctuations during DMPG's anomalous phase transition, there are a number of other interesting contributions to the bending dynamics of highly charged membranes that can be studied in future investigations.

In addition to the membrane softening, both the calculated decrease in *K_A_* and the increase in *d_b_* dispersity measured with SANS point towards an increase in the local thickness fluctuation amplitude in the transition region. While the current results are not quantitative measurements of the thickness fluctuations, the agreement in trends suggests that qualitative information about the amplitude of these fluctuations can be extracted from SANS data when the polydispersity in membrane thickness originates from the time dependent thermal fluctuations and not from static variations in thickness in different parts of the membrane. Moreover, an increase in thickness fluctuation amplitude throughout the anomalous phase transition is particularly relevant to DMPG. These fluctuations have been proposed as a mechanism of stochastic pore formation in lipid membranes, and the increase suggested by the present results may also help form porous and/or perforated bilayers in the DMPG transition region.^79^ Together, the NSE results presented here provide a direct measure of the enhanced local membrane fluctuations and demonstrate how these dynamics are coupled to changes in the bilayer structure than can also affect the macroscopic solution properties.

Finally, the present work highlights that NSE can be a useful tool for measuring the bending moduli of soft membranes when other techniques, such a micropipette aspiration or flicker spectroscopy, cannot be used. While the various techniques are known to provide reliable trends, it is also important to note that different characterization methods are known to give different values of the bending modulus. The *κ* values obtained from flicker spectroscopy on GUVs are typically larger than values obtained from diffuse x-ray scattering,^58^ which may suggest that results are affected by the different length scales probed in the measurement. NSE measurements are performed at the nanometer length scale, similar to x-ray scattering; however, an important distinction is that NSE measures dynamics in the time domain. As such, understanding the dispersion relationships that govern how the dynamics relax are important in interpreting NSE data. The present work calculated *κ* values using the Watson–Brown refinement to the Zilman–Granek model, which inherently assumes that dissipation within the membrane is important at small length- and timescales. Ultimately, the use of several complementary techniques will provide a more complete picture of membrane dynamics and help bridge the molecular and macroscopic scales.

## V. CONCLUSION

The present results nicely fill a gap in our understanding of lipid melting and the anomalous DMPG phase transition. The NSE results provide direct experimental evidence for the increase in local fluctuations and membrane softening during the phase transition. Combining the NSE results with SANS measurements of the bilayer thickness also allowed us to calculate the non-monotonic trends in bilayer area compressibility with temperature. Measuring these enhanced fluctuations in DMPG membranes would have been difficult with other characterization method, making this work an excellent example of the complementarity of NSE to other biophysical characterization techniques. We show that NSE can provide new insights into collective membrane fluctuations, and we hope this work inspires future studies into the dynamics of soft membranes.

## SUPPLEMENTARY MATERIAL

See the supplementary material for ⟨τ⟩ calculated from the NSE data at *q* = 0.3 nm^–1^, 0.8 nm^–1^, and 1 nm^–1^ as well as the diffusion coefficients, *D*, measured with dynamic light scattering (DLS) and used to fit the NSE data with Eq. (1).

## Data Availability

The data that support the findings of this study are available from the corresponding author upon request.
